# Are Differences in Genomic Data Sets due to True Biological Variants or Errors in Genome Assembly: An Example from Two Chloroplast Genomes

**DOI:** 10.1371/journal.pone.0118019

**Published:** 2015-02-06

**Authors:** Zhiqiang Wu, Luke R. Tembrock, Song Ge

**Affiliations:** 1 State Key Laboratory of Systematic and Evolutionary Botany, Institute of Botany, Chinese Academy of Sciences, Beijing, China; 2 Department of Biology, Colorado State University, Fort Collins, Colorado, United States of America; Nanjing Forestry University, CHINA

## Abstract

DNA sequencing has been revolutionized by the development of high-throughput sequencing technologies. Plummeting costs and the massive throughput capacities of second and third generation sequencing platforms have transformed many fields of biological research. Concurrently, new data processing pipelines made rapid *de novo* genome assemblies possible. However, high quality data are critically important for all investigations in the genomic era. We used chloroplast genomes of one *Oryza* species (*O. australiensis*) to compare differences in sequence quality: one genome (GU592209) was obtained through Illumina sequencing and reference-guided assembly and the other genome (KJ830774) was obtained via target enrichment libraries and shotgun sequencing. Based on the whole genome alignment, GU592209 was more similar to the reference genome (*O. sativa*: AY522330) with 99.2% sequence identity (SI value) compared with the 98.8% SI values in the KJ830774 genome; whereas the opposite result was obtained when the SI values in coding and noncoding regions of GU592209 and KJ830774 were compared. Additionally, the junctions of two single copies and repeat copies in the chloroplast genome exhibited differences. Phylogenetic analyses were conducted using these sequences, and the different data sets yielded dissimilar topologies: phylogenetic replacements of the two individuals were remarkably different based on whole genome sequencing or SNP data and insertions and deletions (indels) data. Thus, we concluded that the genomic composition of GU592209 was heterogeneous in coding and non-coding regions. These findings should impel biologists to carefully consider the quality of sequencing and assembly when working with next-generation data.

## Introduction

High-throughput sequencing or next-generation sequencing (NGS) technologies have transformed many fields of biological research: including genetics, phylogenetics, population biology and comparative genomics, by delivering tens of thousands of genome and transcriptome sequences within a short time and with low cost [1, 2]. For example, Illumina announced in 2014 that they could sequence full coverage human genomes for only $1,000 within a few days. At the same time, a diverse array of algorithms was generated to assemble reads from different NGS platforms [3–6]. Despite the advancements brought by NGS technology, biologists remain concerned with obtaining high-quality and high-fidelity data instead of simply acquiring copious quantities of nucleotides. The errors associated with different sequencing platforms and bioinformatic analyses (e.g., reference-guided assemblies) need to be differentiated from true biological variants, such as nucleotide substitutions, insertions or deletions, and large-scale translocations. The errors in sequencing and assembly caused incorrect inferences in genomic analyses such as annotation and downstream analyses [7–10]. For example, Alkan et al. [1] found that *de novo* assembly from a human genome of Han Chinese origin was 16.2% shorter than the reference genome and that 99.1% of the validated duplicated sequences were lost in the comparison to the reference genome. These differences appear inconsequential; however, this translates into more than 2,377 coding exons completely missing from the Han genome. High-quality sequences must be emphasized in combination with high-throughput sequencing, as actively requested by comparative genomic and evolutionary genomic researchers. Zook et al. [11] recently showed that existing sequencing methods and algorithms produced substantial discordance between different bioinformatic pipelines and thus advocated for caution in producing such data sets. Hence, for NGS genome assemblies and downstream comparative analyses, it is paramount to critically assess and compare sequence data to differentiate errors and artifacts from true variants.

Microstructural changes, including insertions and deletions (indels), which frequently occur in intronic and intergenic regions, are just some of the problems biologists face during assembly and mapping of short high-throughput reads [12–14]. Diverse algorithms were developed to tackle the challenge posed in assembling from NGS data sets [14, 16]. Indels are an important class of mutations that not only provide a basis for analytical procedures (i.e., synapomorphies in phylogenetic analyses) but are also linked to genetic diseases [17]. For example, cystic fibrosis, one of the most common genetic diseases in humans, is frequently caused by a single amino acid deletion within the CFTR gene [18]. Indels are often treated as a “fifth base” and occasionally contain a valuable evolutionary signal. In the angiosperms, indels were successfully used to resolve phylogenetic relationships among basal lineages [19] and among closely related taxa [20, 21]. In both crop breeding and population genetics studies, useful molecular markers for the accurate and efficient identification of individuals and populations were indels [22, 23]. Ultimately, the documentation and verification of indels is based on the quality of the assembled genome sequence.

Compared with the gigantic nuclear genome, chloroplast genomes (plastomes) are relatively small, and thus sequencing can be conducted more quickly and at a lower cost. Typically, plastomes exhibit a conserved circular double-stranded DNA arrangement, with sizes that ranged from 115 to 165 kb [24, 25], and the gene content and gene order [26] are highly preserved in the land plants. These features and the high-through sequencing technologies led to an increase in the number of the completed plastomes. Complete plastome sequences from more than 400 species are currently stored in the NCBI database (http://www.ncbi.nlm.nih.gov/genomes/; S1 Table). Publically available plastome sequences such as those stored at NCBI provide a valuable genetic resource for several different types of biological research. First, plastome sequences are a primary source for plant molecular systematic studies [27–31]. The increasing number of complete plant plastome sequences that possess low rates of nucleotide substitutions and structural changes are well suited to resolve the relationships among different plant lineages [30, 32–34]. Second, plastomes of plants are an important resource for DNA barcoding, which is based on sequences from a short and standardized DNA region to identify species [35, 36]. The loci of *matK*, *rbcL*, *atpF-atpH*, *trnH-psbA*, and *psbK-psbI* were used successfully in barcoding efforts to identify species [37–39]. Third, compared with the transformations of the nuclear genome in biotechnology, chloroplast transformations function more effectively [40–42]. The configuration of the transformation vector was primarily based on a similar sequence from the plastome sequence [43, 44]. These applications are all dependent on high quality plastome sequences.

In this study, we compared whether the sequence differences were real variants or rather the result of sequencing or assembly errors. The comparisons were conducted between two published plastomes from two individuals of *Oryza australiensis* (Domin & C.E. Hubb). One plastome (*O. australiensis*: GU592209) was obtained through Illumina sequencing and reference-guided assembly [45] and the other plastome (*O. australiensis*: KJ830774) was completed through the construction of target enrichment libraries and shotgun Sanger sequencing [46]. These two different sequencing and assembling strategies provided the basis for the comparisons. *O. australiensis* is a diploid species from the E-genome group of the rice genus and is an important wild relative to domesticated rice [47–49]. We systematically compared these two plastomes by whole genome alignment, including examination of the sequence identity in both the coding and noncoding regions and the variation in the junction of single copy and repeat copy in the plastome. Additionally, phylogenetic analyses were conducted based on the whole plastome sequence, single nucleotide polymorphisms (SNP) and indels data. We found that the quality of sequences and assemblies from high-throughput genome sequencing deserved special attention.

## Materials and Methods

### Plastome annotation

All eight published plastomes from the *Oryza* genus and an out-group plastome sequence from the species *Leersia tisserantii* (A. Chev. Launert) (the closest relative in the same tribe of Oryzeae) were downloaded from the NCBI database (Table 1). To fully and consistently compare the plastome annotation, DOGMA (Dual OrganellarGenoMe Annotator [50]) was employed for genome annotation, which included the protein-coding genes, transfer RNAs (tRNAs), and ribosomal RNAs (rRNAs). To accurately confirm the start and stop codons and the exon-intron boundaries of genes, the draft annotation was subsequently inspected and adjusted manually based on the published plastomes from the database. Additionally, both tRNA and rRNA genes were identified by BLASTN searches against the same database of plastomes. The tRNAscan-SE 1.21 [51] was also used to further verify the tRNA genes.

**Table 1:** Comparison of the major features of nine chloroplast genomes from the rice tribe (Oryzeae).

Species	Total size		LSC region		IR region		SSC region		GenBank accession no.	Ambiguous Base (N)	Reference
	Length (bp)	GC (%)	Length (bp)	GC (%)	Length (bp)	GC (%)	Length (bp)	GC (%)			
*Oryza sativa* ssp. *Indica*	134,496	39.00	80,553	37.11	20,798	44.35	12,347	33.32	NC_008155	-	[56]
*Oryza nivara*	134,494	39.01	80,544	37.12	20,802	44.35	12,346	33.33	NC_005973	-	[56]
*Oryza sativa* ssp. *Japonica*	134,551	39.00	80,604	37.11	20,802	44.35	12,343	33.37	AY522330	-	[53]
*Oryza rufipogon*	134,557	39.01	80,604	37.11	20,803	44.35	12,347	33.36	NC_022668	-	[57]
*Oryza rufipogon*	134,544	39.00	80,594	37.11	20,802^a^	44.35	12,347	33.33	NC_017835	-	[57]
*Oryza meridionalis*	134,551	39.01	80,606	37.11	20,802	44.35	12,343	33.36	GU592208	-	[45]
*Oryza australiensis*	134,549	38.93	80,614	37.07	20,796	44.36	12,343	33.25	GU592209	177 bp	[45]
*Oryza australiensis*	135,224	38.95	81,074	37.07	20,840	44.33	12,470	33.18	KJ830774	-	[46]
*Leersia tisserantii*	136,551	38.88	81,865	37.01	21,329	44.05	12,027	33.23	JN415112	-	[30]

a: Two IR regions have one base pair difference in this species.

### Differences from comparative chloroplast genomic analysis

To fully compare the complete plastomes of *O. australiensis* isolate 86524 (KJ830774, [46]) and *O. australiensis* isolate 300136 (GU592209, [45]), the mVISTA program was employed in the Shuffle-LAGAN mode [52] to detect whole genome variation. The plastome of *O. sativa* ssp. *Japonica* (AY522330, [53]) was used as a reference. To assess the sequence identity (SI) values of the coding and noncoding regions of the two plastomes (KJ830774 and GU592209), the nucleotide sequences of all protein coding and RNA genes and noncoding sequences were aligned to the reference genome (*O. sativa* ssp. *Japonica*, AY522330) using the ClustalX [54] and adjusted manually, and the SI values were calculated using the BioEdit [55]. The final alignments are shown in the S2 Table.

### Differences from phylogenetic reconstructions using different data sets

To construct and compare the phylogenetic relationships of different data sets, nine published plastomes from the rice tribe (Oryzeae) were downloaded from the NCBI database for use in the analyses (Table 1). In the first phylogenetic analysis, the whole plastome sequence data were used. Based on the conserved structure and gene order of chloroplast genomes [26], the sequence alignments were made in the BioEdit software [55] with the coding gene positions manually inspected (S2 Table). Four methods were employed to construct the phylogenetic trees, including maximum parsimony (MP) implemented with PAUP 4.0b10 [58], maximum likelihood (ML) [59] and neighbor-joining (NJ) with MEGA6 [59], and Bayesian inference (BI) with MrBayes3.1.2 [60]. Using a heuristic search with 1000 random addition sequence replicates, the MP method was executed under tree-bisection-reconnection (TBR) branch-swapping tree search criteria. Parameters for the ML analysis were optimized with a BIONJ tree as a default point with 1000 bootstrap replicates using the Kimura 2-parameter model and the gamma distribution with invariant sites for rate variation. The NJ settings employed 1000 bootstrap replicates using the p-distance model with uniform rates. For the estimation of Bayesian posterior probabilities (PP) in the BI analyses, the MCMC algorithm was run for 1,000,000 generations with 4 incrementally heated chains, starting from random trees and sampling one out of every 100 generations. When the log-likelihood scores stabilized, a consensus tree was calculated after discarding the first 25% of the trees as burn-in.

In the second phylogenetic analysis, only single nucleotide polymorphism (SNP) data were used. The SNP matrix was extracted using the DAMBE software [61] from the aligned whole genome data set used previously (S2 Table). Furthermore, three SNP matrices were built that contained the whole plastome, coding regions or noncoding regions. The neighbor-joining (NJ) and unweighted pair group method with arithmetic mean (UPGMA) methods were used to construct the phylogenetic tree in MEGA6 [59]. Both methods were run using 1000 bootstrap replicates and the p-distance model with uniform rate variation.

In the third analysis, only the indels matrix from noncoding regions was extracted to construct the phylogenetic trees. Microstructural changes such as indels were widely used for resolving phylogenetic relationships [19–21]. The software DnaSP5 [62] was employed to acquire the indels polymorphism using the aligned data from above. The indels data were checked manually to confirm the reliability. All 527 indels sites (S3 Table) were used in the phylogenetic analysis. The indels sites were coded with zero (nongap variant) and one (gap variant). The settings for MP and BI analyses were identical to those used in the whole genome work described above. The neighbor-joining (NJ) tree was resolved in R with the ‘phangorn’ package [63] with 1000 bootstrap replicates.

## Results and Discussion

### Overview of plastome sequencing

**Fig 1:**
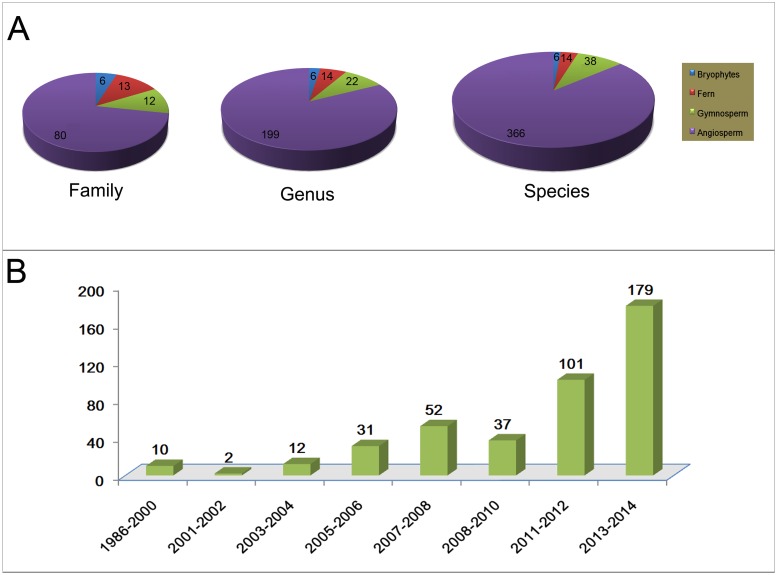
Information from the published chloroplast genomes in land plants, as of February 1, 2014. A. The list of plastomes was acquired from the NCBI Organelle Genome Resources (http://www.ncbi.nlm.nih.gov/genomes/) and related published reports. B. Number of plastomes published since 1986. The year of each genome sequence is according to the release date of its upload to GenBank.

From the time the first two species (*Marchantia polymorpha* L. and *Nicotiana tabacum* L.) plastomes were sequenced [64, 65], over 400 chloroplast genomes of land plants (Fig. 1 and S1 Table) have been published (as of February 2014). Of the over 400 complete plastome sequences, angiosperms were 72.07% of the data set, gymnosperms 10.81%, ferns 11.71%, and bryophytes 5.41% (Fig. 1A). Angiosperm species occupied the dominant priority (Fig. 1A) because the plastomes of most angiosperms are highly conserved in genome size, gene content and gene order [26].

The rapid increase in number of complete plastome sequences is attributed to the advances in sequencing technologies. Before 2005, approximately two dozens plastomes were sequenced. At that time, the chemical method (Gilbert) and the dideoxy nucleotide procedure (Sanger) were the major techniques to sequence plastomes. These methods for sequencing a complete plastome were expensive, slow and laborious [66]. Because of limitations associated with the pre-NGS sequencing techniques, only model species were targeted for complete plastome sequencing. Since the development of the next-generation sequencing (NGS) platforms, the rate and number of sequenced plastomes increased rapidly, and more nonmodel species were sequenced (Fig. 1B). For example, Park et al. [67] was able to fully sequence 36 species in Pinaceae in a single study using the Illumina-Solexa platform. Similarly, Bayly et al. [68] used the Illumina platform to sequence 39 species in the eucalypt group. The unprecedented power of NGS undoubtedly increased the number of finished plastomes. However, the quality and accuracy of plastomes generated from these methods should be viewed with caution. For example, ambiguous bases still remained in the finished genomes, and some inverted repeat regions were of varying lengths (S1 Table). Of 424 plastomes, 51 (12.03%) plastomes contained ambiguous bases regardless of which methods were used to sequence them. Hence, it is imperative to carefully execute quality control on NGS sequence reads as the technology becomes ubiquitous in the biological and medical fields [1, 12].

### Differences from plastome junction boundary

**Fig 2:**
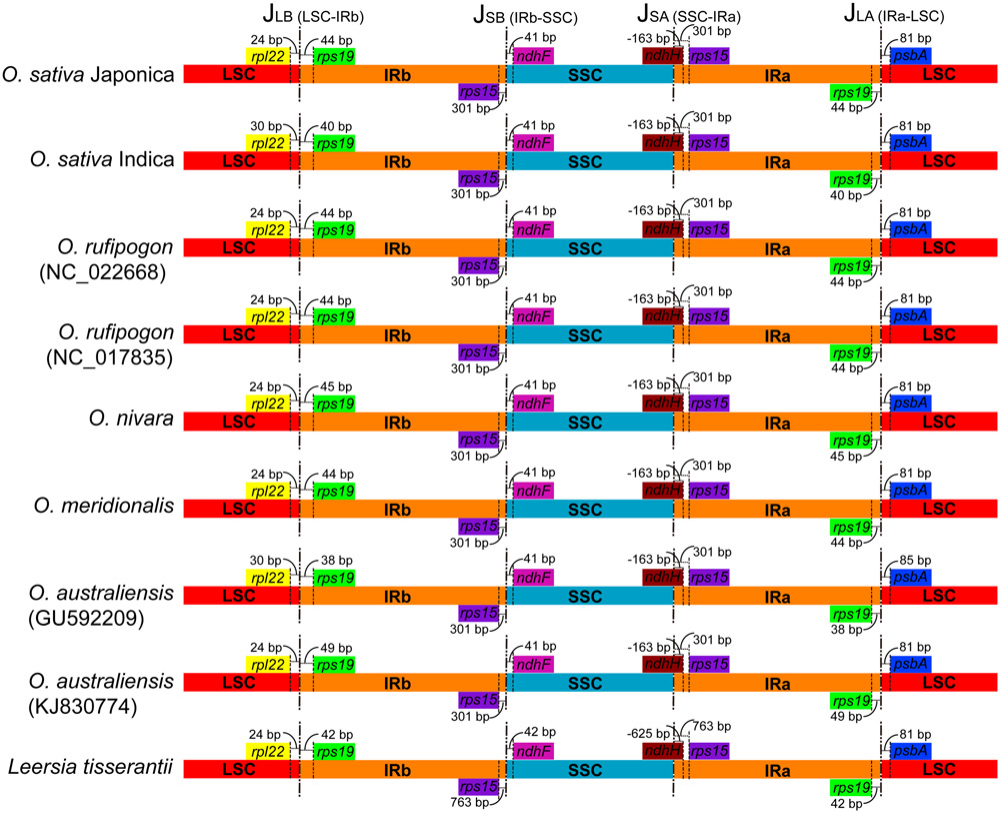
Comparisons of border distances between adjacent genes and junctions of LSC, SSC, and two IR regions among nine rice tribe chloroplast genomes. Boxes above or below the main line indicate the adjacent border genes. The figure is not to scale with sequence length and only shows relative changes at or near the IR/SC borders.

Two inverted repeats (IRs) and two unequal single-copy regions characterized the typical quadripartite structure of plastomes from most land plants [25, 69]. Previous study (e.g., [25]) showed that the extension or contraction of IR regions is one of the major mechanisms causing variation in plastome size [25]. Wang et al. [70] uncovered the dynamics and evolution of the border regions between the two IR regions and the single-copy regions among monocot lineages. Four junctions (J_LA_, J_LB_, J_SA_, and J_SB_) were between the two IRs (IR_A_ and IR_B_) and the two single copy (LSC and SSC) regions (Fig. 2) [70]. We carefully compared the exact IR border positions and the adjacent genes among the eight in-group *Oryza* and the one out-group species (*L. tisserantii*) [30] plastomes (Fig. 2). For J_LA_, it was located between *rps19* and *psbA*. The variation in distances between *rps19* and J_LA_ was from 40 bp to 49 bp; however, the distance between *psbA* and J_LA_ was consistent at 81 bp, except for *O. australiensis* (GU592209) with 38 bp and 85 bp, respectively. For J_LB_, the distance between *rpl22* and J_LB_ varied from 24 bp to 30 bp. When compared with J_LA_ and J_LB_, however, the border regions for J_SA_ and J_SB_ were more conserved. The *ndhH* gene spanned the SSC and IR_A_ region with approximately 163 bp located in the IR region for all eight *Oryza* species. The *ndhF* gene was located in the SSC region, and 41 bp distances were also conserved for all eight *Oryza* species. The same distance was found for the *rps15* gene (301 bp). However, when the out-group species was considered, the main variation was located in the border regions of SSC and IR. For the *ndhH* gene, approximately 625 bp were integrated into IR_A_ region. This 625 bp extension also contributed to the overall size differences between the out-group and the *Oryza* species plastomes [25].

### Comparative differences between the two plastomes

We compared the plastome (*O. australiensis*: GU592209) that was sequenced via Illumina and reference-guided assembly [45], with a plastome (*O. australiensis*: KJ830774) that was completed with target enrichment libraries and shotgun Sanger sequencing [46]. The two published plastomes of *O. australiensis* demonstrated the two different sequencing and assembling strategies and provided an opportunity to compare the sequence quality of the two methods. How to handle the repetitive regions is one of the intractable bottlenecks for practical assembly of next-generation short reads [71], and the same problem was introduced for the reference-guided assembly for *O. australiensis* (GU592209). This might cause some variation for the two inverted repeats and their junction regions. For the plastome of *O. australiensis* (KJ830774), Fosmid libraries were constructed, followed by shearing, cloning, and sequencing. This method was labor-intensive but was shown to be an effective approach for obtaining high quality sequence data [72].

**Fig 3:**
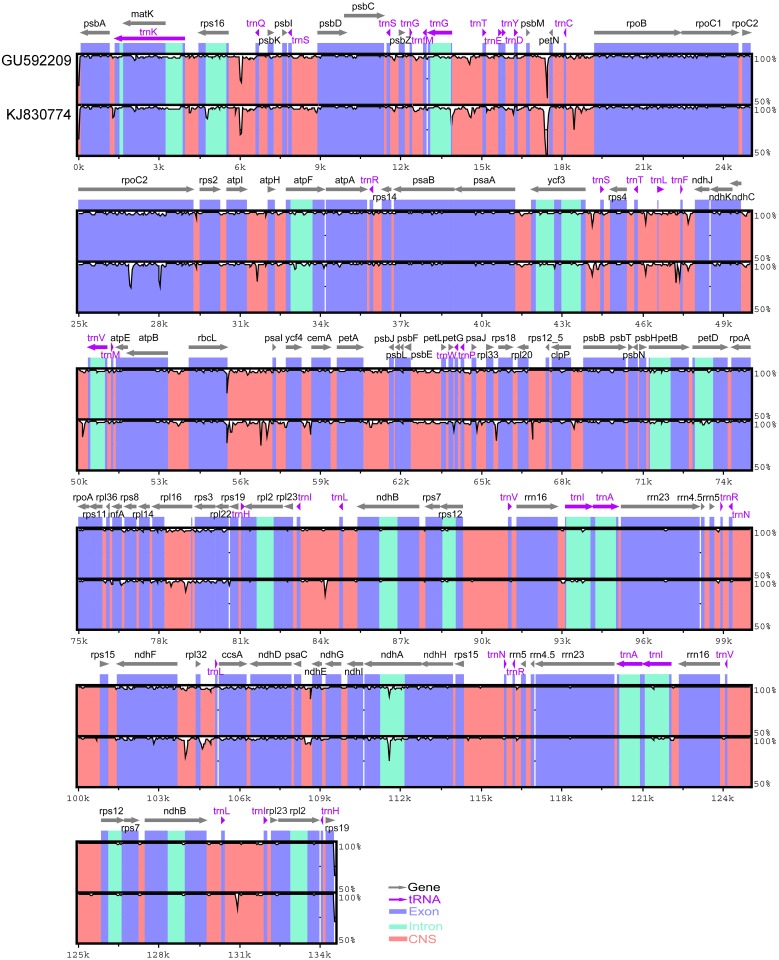
Identity plot that compares the chloroplast genomes of the two *O*. *australiensis* data sets used in this study with *O. sativa* ssp. *Japonica* (AY522330) as the reference sequence. The vertical scale indicates the percentage of identity, ranging from 50% to 100%. The horizontal axis indicates the coordinated base position within the chloroplast genome. Genome regions are color coded as protein-coding, rRNA, tRNA, intron, and conserved noncoding sequences (CNS).

First, the mVISTA program [52] was used to demonstrate the whole genome variation with *O. sativa* ssp. *Japonica* (AY522330) as the reference for comparison with the two plastomes (Fig. 3). As the whole, the organization of the plastome was rather conserved between two individuals, and no translocations or inversions were detected in the architecture of the two genomes. The two IR regions were more conserved than the LSC and SSC regions. However, we found more local variations in *O. australiensis* (KJ830774) than in *O. australiensis* (GU592209). For example, two variations in the *rpoC2* gene were found in KJ830774 but not in GU592209. Many of the intergenic region (*ndhC-trnV*, *rbcL-psaI* and others) variations were found in KJ830774, but no such variation was found in GU592209. The results indicated that the full sequence of GU592209 was more similar to AY522330 and that KJ830774 was more divergent compared with GU592209.

**Fig 4:**
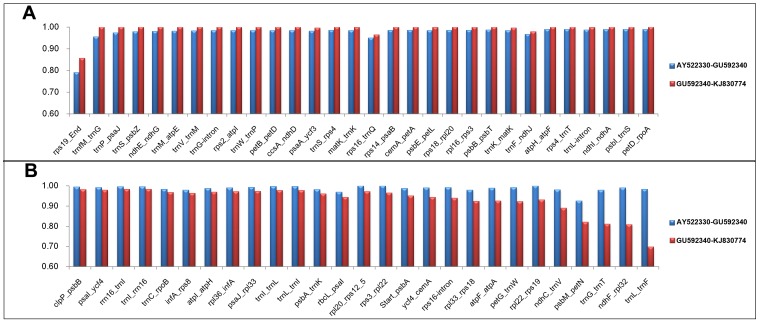
Sequence identity (SI) variations are presented for 149 intergenic sequences (IGS) between *O*. *sativa* ssp. *Japonica* (AY522330) and *O. australiensis* (GU592340) versus between *O. sativa* ssp. *Japonica* (AY522330) and *O. australiensis* (KJ830774). A. 30 IGS regions with SI values GU592209-KJ830774 larger than AY522330-GU592209 values. B. 27 IGS regions with SI values AY522330-GU592209 larger than GU592209-KJ830774 values. The 43 IGS regions with no differences and the 49 IGS regions with less than 1% difference for SI values are not shown.

Second, to further examine the differences of the two individual plastomes, we divided the plastome into individual genes (coding) and intergenic regions (noncoding). For all nine species, 111 genes were annotated, which was the same as other published species [30]. Of these genes, 103 (92.8%) genes were found with 100% sequence identity (SI) between KJ830774 and GU592209. 52 genes were found with 100% SI between GU592209 and AY522330. However, of these 52 genes, 51 genes shared 100% SI among AY522330, GU592209 and KJ830774. Only two genes (*rpl32* and *rpoC2*) were found to have same level of SI between GU592209 and AY522330 compared with KJ830774. From these coding sequence SI results, KJ830774 was more similar to GU592209. However, the intergenic sequences (noncoding regions, IGS) exhibited different trends (Fig. 4). Among 149 IGS, 30 demonstrated high SI (1% to 6.6% difference) in GU592209-KJ830774 compared with AY522330-GU592209, and 27 IGS displayed high SI (1.2% to 28.5% difference) in AY522330-GU592209 compared with GU592209-KJ830774. For the remaining IGS, 43 had no SI difference and 49 showed less than 1% in SI difference. From examination of noncoding regions, GU592209 was more similar to the reference genome (AY522330). We also compared the whole genome SI value and found that GU592209 and AY522330 had 99.2% sequence similarity. However, the similarity was 98.2% for KJ830774 and AY522330. Although GU592209 was published as an unfinished genome (177 ambiguous bases (N)), those ambiguous bases were distributed in 18 different regions with lengths ranging from 1 bp to 45 bp (S3 Table). When we excluded them from analysis, the results were the same as above. Integrating this evidence, GU592209 contained heterogeneity in coding and non-coding regions, and therefore, the assembled plastome for GU592209 might be inaccurate.

### Phylogenetic reconstruction from different data sets

**Fig 5:**
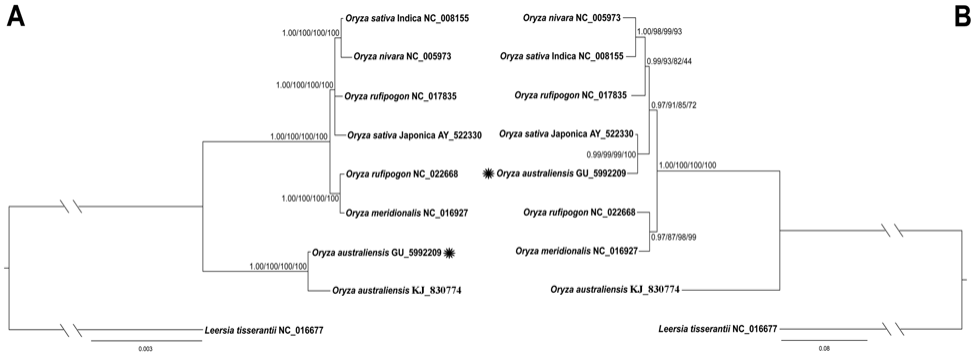
Phylogenetic trees were constructed for nine species from the rice tribe using different methods, and two Bayesian trees are shown for the whole genome sequence and the insertion-deletion data. A. The whole genome sequence data were used with four different methods, Bayesian inference (BI), maximum parsimony (MP), maximum likelihood (ML) and neighbor-joining (NJ). Numbers above the branches are the posterior probabilities for BI and bootstrap values of MP, ML and NJ, respectively. B. The coding data from insertions and deletions (indels) were used with three different methods, Bayesian inference (BI) and maximum parsimony (MP), and two neighbor-joining (NJ) methods, for two different sets of coded data. Numbers above the branches are the posterior probabilities for BI and bootstrap values of MP and NJ. Branch length is proportional to the number of substitutions, as indicated by the scale bar. Stars represent the different positions for *O. australiensis* (GU592340) in the two trees.

From the results described above, we concluded that coding and noncoding regions of *O. australiensis* (KJ830774) and *O. australiensis* (GU592209) might contain different phylogenetic signals. Therefore, the plastome data were divided into 1) the whole genome sequence, 2) three SNPs matrices (extracting all polymorphic sites using the DAMBE software) from the whole plastome, coding or noncoding regions, and 3) indels from noncoding regions to examine our deduction. Different methods were used to construct the phylogenetic trees (Fig. 5).

The whole plastome sequence (S2 Table) and SNP (from whole plastome, coding or noncoding regions) data generated the same phylogenetic tree (Fig. 5A). In the phylogenetic trees from these two types of data sets, *O. australiensis* (KJ830774) and *O. australiensis* (GU592209) formed a single clade with high BI and bootstrap support under the four different methods. Moreover, the tree topology corroborated the relationships inferred from the phylogenetic work conducted by Zou et al. [48]. All the other six *Oryza* species formed one well-supported branch and were from the A-genome and *O. australiensis* was in the E-genome group in the rice genus [47, 48], which evolved in the middle Miocene [49]. The two cultivated and two wild rice individuals formed a well-supported clade; however, individual relationships within this clade could not be fully resolved. This result that concerned the wild and cultivated lineages of rice was similar to that from Waters et al. [57]. However, when we applied our methods for phylogenetic reconstruction using the indels-only data set: *O. australiensis* was resolved on different branches (Fig. 5B). From the indels data, *O. australiensis* (GU592209) was a sister to *O. sativa* ssp. *Japonica* (AY522330) with high BI and bootstrap support, whereas *O. australiensis* (KJ830774) was resolved as a sister to all other *Oryza* species (formed an AA genome clade) in all analyses. From this analysis, the two *O. australiensis* individuals were placed in two different clades. The position of *O. australiensis* (GU592209) did not conform to previously published phylogenies for the group [47, 48] nor was it resolved as sister to the other *Oryza* individuals. However, *O. australiensis* (KJ830774) still remained sister to the remaining *Oryza* species as was found in previous studies [47, 48]. When using the phylogenetic analyses to test for differences between sequencing and alignment methods, we found that *O. australiensis* (GU592209) was heterogeneous in the assembled sequences for coding and noncoding regions.

## Conclusions

With the development of next-generation sequencing technologies, it is now possible to sequence whole nuclear genomes of any species, including the chloroplast genome. However, it is urgent for us to consider the sequencing quality of the NGS data. In this study, we employed the plastomes to carefully compare the quality of chloroplast genomes generated with two different sequencing strategies. Two *O. australiensis* individual plastome sequences were generated. The *O. australiensis* (GU592209) was sequenced using NGS and assembled with a reference genome, whereas *O. australiensis* (KJ830774) was constructed using Fosmid libraries and sequenced with clone sequencing. For the whole genome alignment, *O. australiensis* (GU592209) was more similar to the reference with 99.2% sequence identity than *O. australiensis* (KJ830774) with 98.8% sequence identity. From the sequence analysis, the coding regions of the two individuals contained no differences from the references genome; however, for the intergenic regions, *O. australiensis* (GU592209) was more similar to the reference than *O. australiensis* (KJ830774). The phylogenetic analyses also found that coding and noncoding regions generated two different topologies regarding the replacement of *O. australiensis* (GU592209). From all the analyses, we concluded that the plastome of *O. australiensis* (GU592209) obtained via NGS might be less accurate than the *O. australiensis* (KJ830774) plastome that was generated via Sanger sequencing. Thus, our finding demonstrates the requirement for careful quality control as NGS methods become more prevalent in biological studies.

## Supporting Information

S1 Table 0424 chloroplast genomes downloaded from the NCBI database. (XLSX)

S2 Table The whole genome alignment of plastid genome from nine species. (NEX)

S3 Table Indels code matrix from nine species and 18 regions with N base pair from GU592209. (XLSX)

## References

[1] Alkan C, Sajjadian S, Eichler EE (2011) Limitations of next-generation genome sequence assembly. Nat Methods 8: 61–65. 10.1038/nmeth.1527 21102452 PMC3115693

[2] Steele PR, Hertweck KL, Mayfield D, McKain MR, Leebens-Mack J, et al (2012) Quality and quantity of data recovered from massively parallel sequences: examples in Asparagales and Poaceae. Am J Bot 99: 330–348. 10.3732/ajb.1100491 22291168

[3] Zerbino DR, Birney E (2008) Velvet: Algorithms for de novo short read assembly using de Bruijn graphs. Genome Res 18: 821–829. 10.1101/gr.074492.107 18349386 PMC2336801

[4] Li H, Ruan J, Durbin R (2008) Mapping short DNA sequencing reads and calling variants using mapping quality scores. Genome Res 18: 1851–1858. 10.1101/gr.078212.108 18714091 PMC2577856

[5] Luo R, Liu B, Xie Y, Li Z, Huang W, et al (2012) SOAPdenovo2: an empirically improved memory-efficient short-read de novo assembler. GigaScience 1: 18 10.1186/2047-217X-1-18 23587118 PMC3626529

[6] Bradnam KR, Fass JN, Alexandrov A, Baranay P, Bechner M, et al (2013) Assemblathon 2: evaluating de novo methods of genome assembly in three vertebrate species. GigaScience 2: 10 10.1186/2047-217X-2-10 23870653 PMC3844414

[7] Lunter G, Ponting CP, Hein J (2006) Genome-wide identification of human functional DNA using a neutral indel model. PLoS Comput Biol 2: e5 16410828 10.1371/journal.pcbi.0020005 PMC1326222

[8] Phillippy AM, Schatz MC, Pop M (2008) Genome assembly forensics: Finding the elusive mis-assembly. Genome Biol 9: R55 10.1186/gb-2008-9-3-r55 18341692 PMC2397507

[9] Treangen TJ, Salzberg SL (2011) Repetitive DNA and next-generation sequencing: computational challenges and solutions. Nat Rev Genet 13: 36–46. 10.1038/nrg3117 22124482 PMC3324860

[10] Schatz MC, Witkowski J, McCombie WR (2012) Current challenges in de novo plant genome sequencing and assembly. Genome Biol 13: 243 22546054 10.1186/gb-2012-13-4-243 PMC3446297

[11] Zook JM, Chapman B, Wang J, Mittelman D, Hofmann O, et al (2014) Integrating human sequence data sets provides a resource of benchmark SNP and indel genotype calls. Nat Biotechnol 32: 246–251. 10.1038/nbt.2835 24531798

[12] Meader S, Hillier LW, Locke D, Ponting CP, Lunter G (2010) Genome assembly quality: assessment and improvement using the neutral indel model. Genome Res. 20: 675–684. 10.1101/gr.096966.109 20305016 PMC2860169

[13] Mahmud MP, Wiedenhoeft J, Schliep A (2012) Indel-tolerant read mapping with trinucleotide frequencies using cache-oblivious kd-trees. Bioinformatics 28 (18): i325–i332. 10.1093/bioinformatics/bts380 22962448 PMC3436807

[14] Grimm D, Hagmann J, Koenig D, Weigel D, Borgwardt K (2013) Accurate indel prediction using paired-end short reads. BMC Genomics 14: 132 10.1186/1471-2164-14-132 23442375 PMC3614465

[15] Krawitz P, Rodelsperger C, Jager M, Jostins L, Bauer S, et al (2010) Microindel detection in short-read sequence data. Bioinformatics 26: 722–729. 10.1093/bioinformatics/btq027 20144947

[16] Li S, Li R, Li H, Lu J, Li Y, et al (2013) SOAPindel: Efficient identification of indels from short paired reads. Genome Res. 23: 195–200. 10.1101/gr.132480.111 22972939 PMC3530679

[17] Ball EV, Stenson PD, Abeysinghe SS, Krawczak M, Cooper DN, et al (2005) Microdeletions and microinsertions causing human genetic disease: Common mechanisms of mutagenesis and the role of local DNA sequence complexity. Hum Mutat 26: 205–213. 16086312 10.1002/humu.20212

[18] Collins FS, Drumm ML, Cole JL, Lockwood WK, VandeWoude GF, et al (1987) Construction of a general human chromosome jumping library, with application to cystic fibrosis. Science 235: 1046–1049. 2950591 10.1126/science.2950591

[19] Graham SW, Reeves PA, Burns ACE, Olmstead RG (2000) Microstructural changes in non-coding DNA: interpretation, evolution and utility of indels and inversions in basal angiosperm phylogenetic inference. Int J Plant Sci 161: S83–S96.

[20] Kelchner SA (2000) The evolution of non-coding chloroplast DNA and its application in plant systematics. Ann MO Bot Gard 87: 499–527.

[21] Ingvarsson PK, Ribstein S, Taylor DR (2003) Molecular evolution of insertions and deletion in the chloroplast genome of *Silene* . Mol Biol Evol 20: 1737–1740. 12832644 10.1093/molbev/msg163

[22] Väli Ü, Brandström M, Johansson M, Ellegren H (2008) Insertion-deletion polymorphisms (indels) as genetic markers in natural populations. BMC Genetics 9: 8 10.1186/1471-2156-9-8 18211670 PMC2266919

[23] Lu BR, Cai XX, Jin X (2009) Efficient indica and japonica rice identification based on the InDel molecular method: Its implication in rice breeding and evolutionary research. Prog Nat Sci 19: 1241–1252.

[24] Palmer JD (1985) Comparative organization of chloroplast genomes. Ann Rev Genet 19: 325–354. 3936406 10.1146/annurev.ge.19.120185.001545

[25] Ravi V, Khurana JP, Tyagi AK, Khurana P (2008) An update on chloroplast genomes. Plant Syst Evol 271: 101–122.

[26] Wicke S, Schneeweiss GM, dePamphilis CW, Müller KF, Quandt D (2011) The evolution of the plastid chromosome in land plants: gene content, gene order, gene function. Plant Mol Bio 76: 273–297. 10.1007/s11103-011-9762-4 21424877 PMC3104136

[27] Shaw J, Lickey EB, Beck JT, Farmer JB, Liu W, et al (2005) The tortoise and the hare II: Comparison of the relative utility of 21 non-coding chloroplast DNA sequences for phylogenetic analysis. Am J Bot 92: 142–166. 10.3732/ajb.92.1.142 21652394

[28] Wang L, Qi XP, Xiang QP, Heinrichs J, Schneider H, et al (2010a) Phylogeny of the paleotropical fern genus *Lepisorus* (Polypodiaceae, Polypodiopsida) inferred from four chloroplast genome regions. Mol Phylogenet Evol 54(1): 211–225. 10.1016/j.ympev.2009.08.032 19737617

[29] Wang L, Wu ZQ, Xiang QP, Heinrichs J, Schneider H, et al (2010b) A molecular phylogeny and a revised classification of tribe Lepisoreae (Polypodiaceae) based on an analysis of four plastid DNA regions. Bot J Linn Soc 162(1): 28–38.

[30] Wu ZQ, Ge S (2012) Phylogeny of the BEP clade in grasses revisited: evidence from whole genome sequences of chloroplast. Mol Phylogenet Evol 62: 573–578. 10.1016/j.ympev.2011.10.019 22093967

[31] Middleton CP, Senerchia N, Stein N, Akhunov ED, Keller B, et al (2014) Sequencing of Chloroplast Genomes from Wheat, Barley, Rye and Their Relatives Provides a Detailed Insight into the Evolution of the Triticeae Tribe. PLoS ONE 9(3): e85761 10.1371/journal.pone.0085761 24614886 PMC3948623

[32] Moore MJ, Bell CD, Soltis PS, Soltis DE (2007) Using plastid genome-scale data to resolve enigmatic relationships among basal angiosperms. Proc Natl Acad Sci USA 104: 19363–19368. 18048334 10.1073/pnas.0708072104 PMC2148295

[33] Jansen RK, Cai Z, Raubeson LA, Daniell H, dePamphilis CW, et al (2007) Analysis of 81 genes from 64 plastid genomes resolves relationships in angiosperms and identifies genome-scale evolutionary patterns. Proc Natl Acad Sci USA 104: 19369–19374. 18048330 10.1073/pnas.0709121104 PMC2148296

[34] Moore MJ, Soltis PS, Bell CD, Burleigh JG, Soltis DE (2010) Phylogenetic analysis of 83 plastid genes further resolves the early diversification of eudicots. Proc Natl Acad Sci USA 107: 4623–4628. 10.1073/pnas.0907801107 20176954 PMC2842043

[35] CBOL Plant Working Group (2009) A DNA barcode for land plants. Proc Natl Acad Sci USA 106: 12794–12797. 10.1073/pnas.0905845106 19666622 PMC2722355

[36] Group CPB, Li DZ, Gao LM, Li HT, Wang H, et al (2011) Comparative analysis of a large dataset indicates that internal transcribed spacer (ITS) should be incorporated into the core barcode for seed plants. Proc Natl Acad Sci USA 108: 19641–19646. 10.1073/pnas.1104551108 22100737 PMC3241788

[37] Pennisi E (2007) Taxonomy. Wanted: A barcode for plants. Science 318:190–191. 17932267 10.1126/science.318.5848.190

[38] Kress WJ, Erickson DL (2007) A two-locus global DNA barcode for land plants: the coding *rbcL* gene complements the non-coding *trnH-psbA* spacer region. PLoS ONE 2: e508 17551588 10.1371/journal.pone.0000508 PMC1876818

[39] Ledford H (2008) Botanical identities: DNA barcoding for plants comes a step closer. Nature 451: 616 10.1038/451616b 18256630

[40] Bock R (2007) Plastid biotechnology: prospects for herbicide and insect resistance, metabolic engineering and molecular farming. Curr Opin Biotechnol 18: 100–106. 17169550 10.1016/j.copbio.2006.12.001

[41] Meyers B, Zaltsman A, Lacroix B, Kozlovsky SV, Krichevsky A (2010) Nuclear and plastid genetic engineering of plants: comparison of opportunities and challenges. Biotechnol Adv 28: 747–756. 10.1016/j.biotechadv.2010.05.022 20685387

[42] Cui C, Song F, Tan Y, Zhou X, Zhao W, et al (2011) Stable chloroplast transformation of immature scutella and inflorescences in wheat (*Triticum aestivum* L.). Acta Biochim Biophys Sin 43: 284–91. 10.1093/abbs/gmr008 21343162

[43] Cheng L, Li HP, Qu B, Huang T, Tu JX, et al (2010) Chloroplast transformation of rapeseed (*Brassica napus*) by particle bombardment of cotyledons. Plant Cell Rep 29: 371–381. 10.1007/s00299-010-0828-6 20179937

[44] Day A, Goldschmidt-Clermont M (2011) The chloroplast transformation toolbox: selectable markers and marker removal. Plant Biotechnol J 9: 540–553. 10.1111/j.1467-7652.2011.00604.x 21426476

[45] Nock CJ, Waters DLE, Edwards MA, Bowen SG, Rice N, et al (2011) Chloroplast genome sequences from total DNA for plant identification. Plant Biotechnol J 9: 328–333. 10.1111/j.1467-7652.2010.00558.x 20796245

[46] Wu ZQ, Ge S (2014) The whole chloroplast genome of wild rice (*Oryza australiensis*). Mitochondrial DNA (Online, 10.3109/19401736.2014.928868) 24960559

[47] Ge S, Sang T, Lu BR, Hong DY (1999) Phylogeny of rice genomes with emphasis on origins of allotetraploid species. Proc Natl Acad Sci USA 96: 14400–14405. 10588717 10.1073/pnas.96.25.14400 PMC24448

[48] Zou XH, Zhang FM, Zhang JG, Zang LL, Tang L, et al (2008) Analysis of 142 genes resolves the rapid diversification of the rice genus. Genome Biol 9: R49 10.1186/gb-2008-9-3-r49 18315873 PMC2397501

[49] Zou XH, Yang Z, Doyle JJ, Ge S (2013) Multilocus estimation of divergence times and ancestral effective population sizes of *Oryza* species and implications for the rapid diversification of the genus. New Phytol 198: 1155–1164. 10.1111/nph.12230 23574344

[50] Wyman SK, Jansen RK, Boore JL (2004) Automatic annotation of organellar genomes with DOGMA. Bioinformatics 20: 3252–3255. 15180927 10.1093/bioinformatics/bth352

[51] Schattner P, Brooks AN, Lowe TM (2005) The tRNAscan-SE, snoscan and snoGPS web servers for the detection of tRNAs and snoRNAs. Nucleic Acids Res 33: W686–W689. 15980563 10.1093/nar/gki366 PMC1160127

[52] Frazer KA, Pachter L, Poliakov A, Rubin EM, Dubchak I (2004) VISTA: computational tools for comparative genomics. Nucleic Acids Res 32: W273–W279. 15215394 10.1093/nar/gkh458 PMC441596

[53] Tang J, Xia H, Cao M, Zhang X, Zeng W, et al (2004) A comparison of rice chloroplast genomes. Plant Physiol 135: 412–420. 15122023 10.1104/pp.103.031245 PMC429394

[54] Thompson JD, Gibson TJ, Plewniak F, Jeanmougin F, Higgins DG (1997) The CLUSTAL_X windows interface: flexible strategies for multiple sequence alignment aided by quality analysis tools. Nucleic Acids Res 25: 4876–4882. 9396791 10.1093/nar/25.24.4876 PMC147148

[55] Hall TA (1999) BioEdit: a user-friendly biological sequence alignment editor and analysis program for Windows 95/98/NT. Nucleic Acids Symp Ser 41: 95–98.

[56] Shahid MM, Nishikawa T, Fukuoka S, Njenga PK, Tsudzuki T, et al (2004) The complete nucleotide sequence of wild rice (*Oryza nivara*) chloroplast genome: first genome wide comparative sequence analysis of wild and cultivated rice. Gene 340(1): 133–9. 15556301 10.1016/j.gene.2004.06.008

[57] Waters DLE, Nock CJ, Ishikawa R, Rice N, Henry RJ (2012) Chloroplast genome sequence confirms distinctness of Australian and Asian wild rice. Ecol Evol 2: 211–217. 10.1002/ece3.66 22408737 PMC3297189

[58] Swofford DL (2002) PAUP*, Phylogenetic Analysis Using Parsimony (* and Other Methods). Sinauer Associates, Sunderland, Massachusetts.

[59] Tamura K, Stecher G, Peterson D, Filipski A, Kumar S (2013) MEGA6: Molecular Evolutionary Genetics Analysis version 6.0 Mol Biol Evol 30: 2725–2729. 10.1093/molbev/mst197 24132122 PMC3840312

[60] Ronquist F, Huelsenbeck JP (2003) MrBAYES 3, Bayesian phylogenetic inference under mixed models. Bioinformatics 19: 1572–1574. 12912839 10.1093/bioinformatics/btg180

[61] Xia X, Xie Z (2001) DAMBE, software package for data analysis in molecular biology and evolution. J Hered 92: 371–373. 11535656 10.1093/jhered/92.4.371

[62] Librado P, Rozas J (2009) DnaSP v5: A software for comprehensive analysis of DNA polymorphism data. Bioinformatics 25: 1451–1452. 10.1093/bioinformatics/btp187 19346325

[63] Schliep K (2011) phangorn: phylogenetic analysis in r. Bioinformatics 27:592–593. 10.1093/bioinformatics/btq706 21169378 PMC3035803

[64] Ohyama K, Fukuzawa H, Kohchi T, Shirai H, Sano T, et al (1986) Chloroplast gene organization deduced from complete sequence of liverwort *Marchantia polymorpha* chloroplast DNA. Nature 322: 572–574.

[65] Shinozaki K, Ohme M, Tanaka M, Wakasugi T, Hayashida N, et al (1986) The complete nucleotide sequence of the tobacco chloroplast genome: its gene organization and expression. EMBO J 5: 2043–2049. 16453699 10.1002/j.1460-2075.1986.tb04464.x PMC1167080

[66] Sugiura M (2003) History of chloroplast genomics. Photosynth Res 76: 371–377. 16228593 10.1023/A:1024913304263

[67] Parks M, Cronn R, Liston A (2009) Increasing phylogenetic resolution at low taxonomic levels using massively parallel sequencing of chloroplast genomes. BMC Biology 7: 84 10.1186/1741-7007-7-84 19954512 PMC2793254

[68] Bayly MJ, Rigault P, Spokevicius A, Ladiges PY, Ades PK, et al (2013) Chloroplast genome analysis of Australian eucalypts—Eucalyptus, Corymbia, Angophora, Allosyncarpia and Stockwellia (Myrtaceae). Mol Phylogenet Evol 69(3): 704–16. 10.1016/j.ympev.2013.07.006 23876290

[69] Raubeson LA, Jansen RK (2005) Chloroplast genomes of plants In:Henry RJ ed. Plant diversity and evolution: genotypic and phenotypic variation in higher plants. Wallingford: CABI Publishing 45–68.

[70] Wang RJ, Cheng CL, Chang CC, Wu CL, Su TM, et al (2008) Dynamics and evolution of the inverted repeat-large single copy junctions in the chloroplast genomes of monocots. BMC Evol Biol 8: 36 10.1186/1471-2148-8-36 18237435 PMC2275221

[71] Zhang W, Chen J, Yang Y, Tang Y, Shang J, et al (2011) A Practical Comparison of De Novo Genome Assembly Software Tools for Next-Generation Sequencing Technologies. PLoS ONE 6(3): e17915 10.1371/journal.pone.0017915 21423806 PMC3056720

[72] Jansen RK, Raubeson LA, Boore JL, dePamphilis CW, Chumley TW, et al (2005) Methods for obtaining and analyzing whole chloroplast genome sequences. Methods Enzymol 395: 348–384. 15865976 10.1016/S0076-6879(05)95020-9

